# Normal Weight, Overweight and Obesity Conditions Associated to Prostate Neoplasm Stages—A Systematic Review and Meta-Analysis

**DOI:** 10.3390/biomedicines13051182

**Published:** 2025-05-13

**Authors:** Elsa Vitale, Alessandro Rizzo, Kurvatteppa Halemani, Asha P. Shetty, Omar Cauli, Francesco Massari, Matteo Santoni

**Affiliations:** 1 Medical Thoracic Oncology Unit, IRCCS Istituto Tumori “Giovanni Paolo II”, 70124 Bari, Italy; vitaleelsa00@gmail.com; 2 S.S.D. C.O.r.O. Bed Management Presa in Carico, TDM, IRCCS Istituto Tumori “Giovanni Paolo II”, Viale Orazio Flacco 65, 70124 Bari, Italy; 3 College of Nursing, All India Institute of Medical Sciences (AIIMS), Raebareli 229405, Uttar Pradesh, India; halemani@aiimsrbl.edu.in; 4 College of Nursing, All India Institute of Medical Sciences (AIIMS), Bhubaneshwar 751019, Odhisa, India; con_asha@aiimsbhubaneswar.edu.in; 5 Nursing Department, Faculty of Nursing and Podiatrics & Frailty and Cognitive Impairment Organized Group (FROG), University of Valencia, 46010 Valencia, Spain; 6 Medical Oncology, IRCCS Azienda Ospedaliero-Universitaria di Bologna, 40138 Bologna, Italy; fmassari79@gmail.com; 7 Department of Medical and Surgical Sciences (DIMEC), University of Bologna, 40138 Bologna, Italy; 8 Oncology Unit, Macerata Hospital, 62100 Macerata, Italy; matteo.santoni82@gmail.com

**Keywords:** association, body mass index, neoplasm staging, prostatic neoplasms

## Abstract

**Background/Objective:** Prostate cancer (PCa) represents the second-most common cancer among men worldwide. Obesity is generally considered as a risk factor for cancer and it has been associated with a 20–30% increased risk of PCa death. The present systematic review and meta-analyses aimed to highlight any existing trends between prostate neoplasm stages according to normal weight, overweight and obesity conditions. **Methods:** All interventional records such as randomized clinical trials, quasi-experimental studies and observational studies were included in the present systematic review and meta-analysis which reported PCa stages according to Gleason (GS) or TNM scores according to the BMI-related incidence, as normal weight, overweight and obesity groups. **Results:** Twenty-nine studies were included in the present study. As regards the GS scoring system, 1.09% of high grade in GS was reported among PCa normal weights. Among PCa overweights, 0.98% of low grade was registered in GS. The same trend was recorded among obese PCa patients, since 0.79% of low grade in GS was also registered. As regards TNM scores, both normal weight, overweight and obese PCa patients registered a significant incidence in non-advanced TNM score, without any significant differences considering higher TNM assessments. **Conclusions:** Although the literature seemed to be more in favor of associations between BMI and GS, no specific mechanisms were highlighted between obesity and PCa progression. In this regard, the low androgen microenvironment in obese men could play an important role, but further studies will be necessary in this direction.

## 1. Introduction

Prostate cancer (PCa) represents the second most frequent cancer and the fifth cause of death among men all around the world, encountering more than 1,460,000 estimated cases and 396,000 deaths in 2022 [1], until 2040 encountering around 2.4 million cases and 712,000 deaths, also due to aging and increasing worldwide population [2]. In Italy, prostate cancer is the most frequent malignancy in men (19.8% of all cancers in men), with an estimated 41,100 new diagnoses in 2023 and an estimated 8200 deaths in 2022. Its incidence increases with advancing age, predominantly affecting males after age 50 with a peak incidence around age 70. The prognosis of this cancer depends on a number of factors and, in particular, on the extent of the neoplasm at the time of diagnosis and the age of the patient [3]. Thanks to surgery improvements and treatment approaches, PCa patients have ameliorated both their duration and quality of life [4,5].

Obesity has been associated with a 20–30% increased risk of PCa death [6]. However, there is very little evidence suggesting a linkage between body mass index (BMI) and PCa [7,8]. Conversely, further evidence suggests an increased risk of PCa or death among patients with a high BMI [9,10].

PCa diagnosis is performed thanks to prostate biopsy and histology, which give important biochemical characteristics of the disease. The Gleason system (GS) represents a histologic scoring system for PCa through structural characteristics [11]. In GS, histologic features defined as grades are recognized, which vary from GS grade 1, indicating the well-differentiated with the best prognosis to GS grade 5, indicating the grimmest prognosis [11,12]. The GS score varies from 2 to 10 and indicates the level of aggressiveness of PCa [11,12,13].

Another cancer scoring system is the TNM classification, which suggests prompt information on cancer extension for its general control [14]. The TNM system aims to control and surveil the cancer [9], helping clinicians in their clinical care and decision making [9]. Evidence suggested that obesity is linked to the frequency of cancers, unfortunate treatment goals and elevated death rates [15,16]. Especially, obesity has been suggested to have a positive and direct correlation with the PCa aggressiveness with also higher GS scores [17,18,19]. Greater BMI values supply a more advantageous microenvironment for PCa onset and development, which embrace dysfunctions in the endocrine system, such as testosterone, estrogen and insulin-like growth factor-I serum levels [20,21]. Particularly, PCa interferes with adipose metabolism and testosterone, highlighting a mutual relationship between periprostatic adipose tissue and cancers [22]. PCa also modulates the leptin secretion linked to the quality of periglandular adipose tissue [23]. Adipocytes and PCa cells are also associated with paracrine cytokines inducing lipolysis in adipocytes and enhancing free fatty acids secretion [24].

Dyslipidemia and saturated fatty acids assumptions seem to be associated with an increased recurrence and risk of death in PCa men [25,26] and are involved in producing numerous elements that enhance PCa cell proliferation and disease advancement, embracing extracellular vesicles [27], pro-inflammatory cytokines [28] and other adipokines [29]. Obesity directly modifies cytokines secretion in adipocytes, impacting cancer growth and progression and enhancing tumor survival [30].

The present systematic review and meta-analyses aimed to highlight any existing trends between prostate neoplasm stages according to normal weight, overweight and obesity conditions.

## 2. Materials and Methods

### 2.1. Methodological Research

The present systematic review and meta-analysis was carried out thanks to the Preferred Reporting Items for Systematic Reviews and Meta-analysis (PRISMA) [31]. The protocol was registered with PROSPERO no. CRD42024580302. Keywords were searched through the MeSH terminology and mixed thanks to Boolean operators (Table 1).

Additionally, a research question was formulated using the PIO methodology (Table 2).

### 2.2. Inclusion and Exclusion Criteria

To include a more extensive number of studies, we included all interventional records of specifically randomized clinical trials, quasi-experimental studies and also observational ones. All the selected studies reported PCa stages according to Gleason or TNM scores according to the BMI-related incidence, as normal weight, overweight and obesity groups. Thus, only frequencies and percentages in BMI-related groups according to the PCa stages were collected. Finally, all records written in English language and available in their full text versions were included. On the other hand, records including pediatric patients or healthy participants were excluded for further analysis.

### 2.3. Manuscripts Selection

At the beginning, articles were recognized thanks to systematic research in Embase, PubMed, Scopus and Web of Science databases and a reference management software uploaded full-text versions and removed duplicates. Two independent reviewers (E.V. and A.R.) read the title and abstract of the selected manuscripts, and those which were classified as unsuitable were removed for further readings. Then, screened records were uploaded and assessed more deeply for their potential eligibility. Any disagreements were discussed and resolved clarifying any doubts and reaching consensus. If the disagreement persisted, another reviewer was consulted (K.H.) and a final decision was reached. Data were extracted including study characteristics, as author, year of publication, aim, design, sample size, cancer stage and incidence of PCa stages according to patients’ BMI conditions.

### 2.4. Selected Records

**Figure 1:**
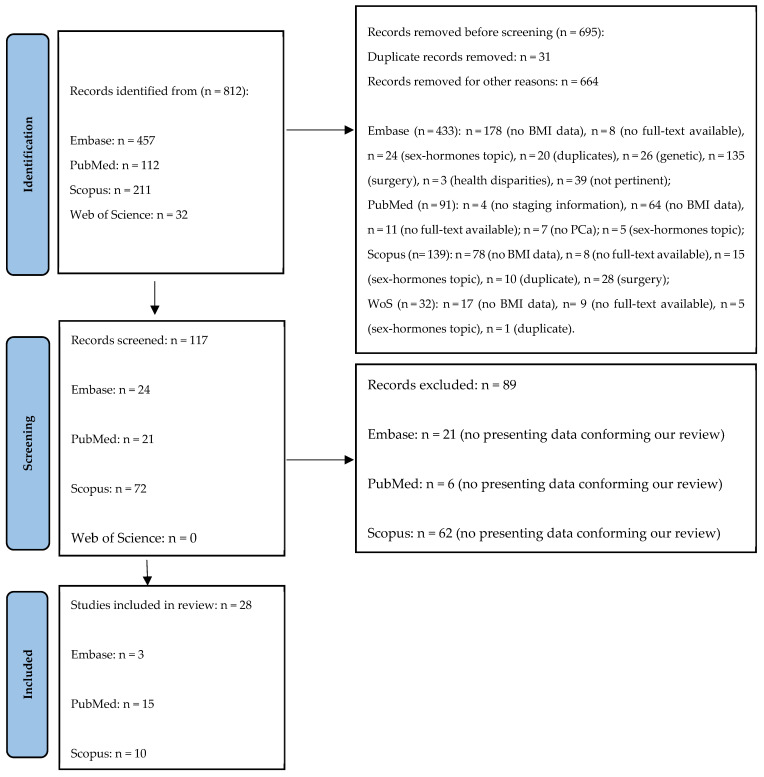
The Prisma flow diagram.

A total of 812 records were identified, specifically 457 from Embase, 112 from PubMed, 211 from Scopus and 32 from Web of Science. Of these, 695 records were removed, as 31 were duplicates and 664 were excluded for other reasons, as shown in Figure 1. From the remaining 111 records, a further 88 were excluded and a total of 29 records were finally included in the present study (Figure 1).

### 2.5. Interventions and Outcomes

The systematic review and meta-analysis included all studies among prostatic cancer patients both assessing the incidence of cases of prostate cancer and its relating staging according to Gleason and TNM score, among normal weight, overweight and obese patients.

Staging was based on the metastases at the tumor node (TNM) classification representing a scoring tool in PCa according to its extension. Specifically, in the initial stage, at the precancerous stage, the tumor was classified at stage T1 and progressively to the T2 stage, in which cancer cells started to rapidly grow and differentiate in tumor forms until reaching stages T3 and T4, in which cancer cells spread to the PCa microenvironments in their tissues and lymph nodes [32]. In the present systematic review and meta-analysis, we gathered the first and the second stage of TNM in the non-advanced stage and the third and the fourth stage of TNM in the advanced stage. Gleason score was assessed thanks to the International Society of Urological Pathology modified Gleason grading system that recommended this scoring system [33], which corresponded well with patient prognosis, and it seemed to be very easy to use in clinical practice [33]. Substantially, a Gleason score less than 6 was associated with a low grade of PCa and a Gleason score more than 7 was associated with high-grade prostate cancer [34]. Finally, body mass index (BMI) was assessed as body weight divided by the square of the height (kg/m^2^) and it was grouped into three main groups, as normal weight (BMI ≤ 24.99), overweight (25.00 ≤ BMI ≥ 29.99) and obese (BMI ≥ 30.00) groups according to the classification of obesity of the World Health Organization [35].

### 2.6. Quality Assessment and Risk of Bias

**Figure 2:**
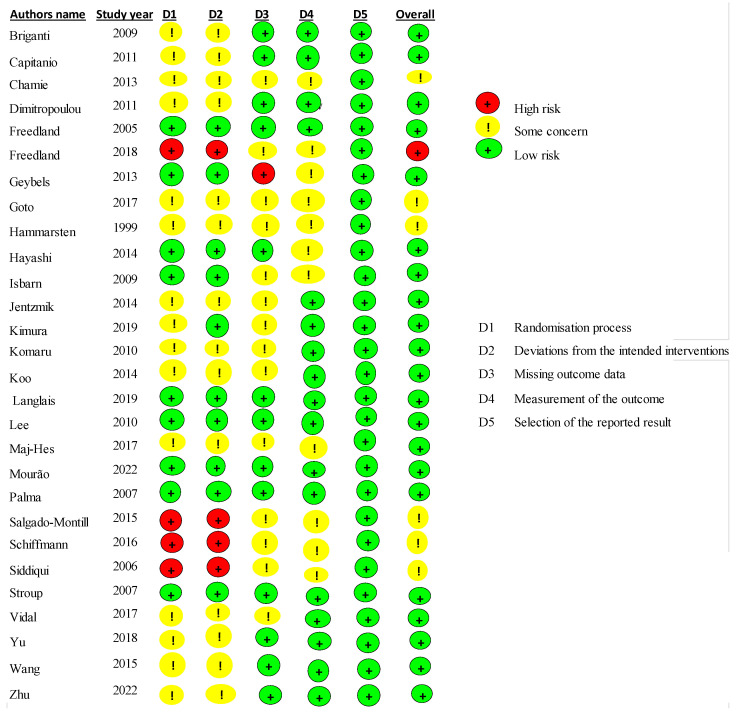
Risk of bias summary of the included studies [37,38,39,40,41,42,43,44,45,46,47,48,49,50,51,52,53,54,55,56,57,58,59,60,61,62,63,64].

Publication bias was evaluated thanks to the risk bias tool which displayed bias risk assessments as well as underlying bias due to the randomization process, bias due to deviations from intended interventions, bias due to missing outcome data and in their related assessments and bias in selection of the reporting approaches [36] (Figure 2).

For each study included in the present review, the authors compared their judgements and then reached these assessments. Overall, the given agreements had low risk for most of the included studies (only 5 out 29 studies had some item of high risk).

### 2.7. Data Analysis

This systematic review and meta-analysis aimed to assess the risk of prostate cancer among normal weight, overweight and obese patients. The body weight was categories-based BMI scores, whereas TNM and Gleason grading were performed as low and high grades. The retrieved data from the original studies were manually entered into Microsoft Excel. Heterogeneity among studies was analyzed using the chi-square test (χ^2^) with 95% (*p* < 0.05) and magnitude of heterogenicity between studies was determined using the I^2^ test values and categorized as high (>75%), medium (50–75%) and low (<50%) heterogeneity. To balance the observed heterogenicity, we employed the random effects model. The effect size for the dichotomous variable was expressed as relative risk, also known as risk ratio, with 95% confidence interval (CI). The pooled data were analyzed using RevMan (Version 5.4. Copenhagen: the Cochrane Collaboration, 2020).

## 3. Results

### 3.1. Selected Studies

Twenty-nine studies were included in the present study as meeting the above-mentioned inclusion criteria [34,37,38,39,40,41,42,43,44,45,46,47,48,49,50,51,52,53,54,55,56,57,58,59,60,61,62,63,64]. Table 3 shows all the main characteristics for each study included in the present systematic review and meta-analysis, specifically study design and PCa assessment, whether it included GS or TNM or both, the aim of the study, the sample size and the achieved findings.

### 3.2. Meta-Analysis According to Gleason Scores

**Figure 3:**
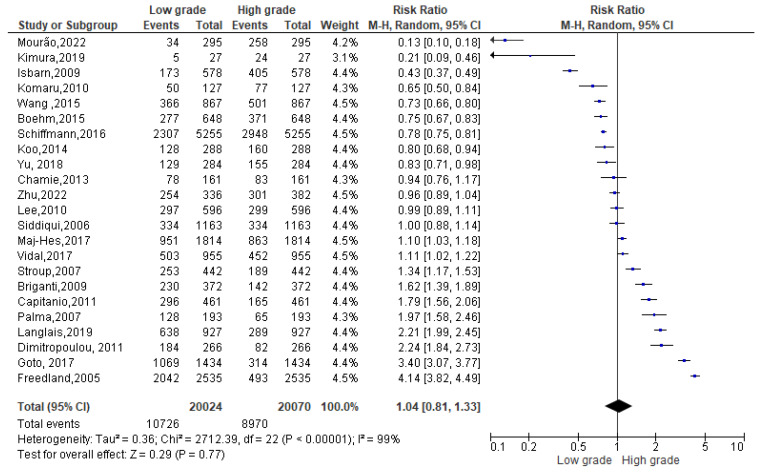
Gleason scores in normal weight PCa patients [34,37,38,39,40,42,44,47,49,50,51,52,53,54,55,56,58,59,60,61,62,63,64].

A total of 57,554 events were registered. Of these, 19,460 reported a normal weight condition, 11,389 an overweight one and 26,705 an obese condition. Among normal weight events reported (Figure 3), 10,791 events belonged to a Gleason low-grade score and 8669 to a Gleason high-grade one. Since the Cochrane Q test showed high heterogeneity (Tau^2^ = 0.36 and *Chi*^2^ = 2712.39 with *I*^2^ = 99%), PCa patients recording a normal weight condition also registered 1.04% of high grade in Gleason score, at 95% confidence intervals [1.04 (0.81,1.33) *p* = 0.00] (Figure 3).

**Figure 4:**
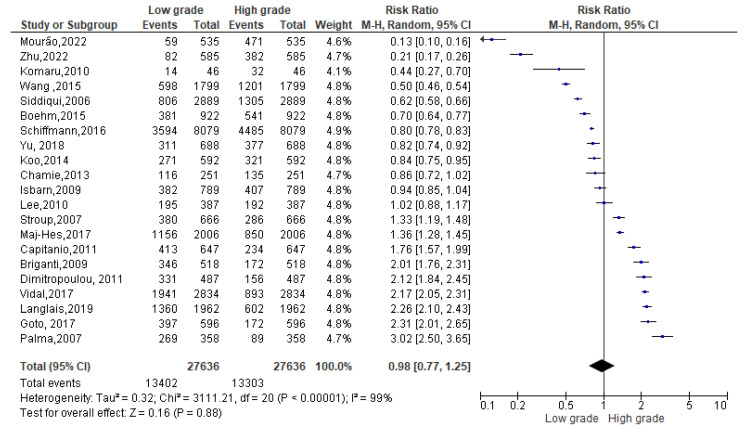
Gleason scores in overweight PCa patients [34,37,38,39,40,44,47,50,51,52,53,54,55,56,58,59,60,61,62,63,64].

Among 26,705 overweight events reported (Figure 4), 13,402 events belonged to a Gleason low-grade score and 13,303 to a Gleason high-grade one. Since the Cochrane Q test showed high heterogeneity (*Tau*^2^ = 0.32 and *Chi*^2^ = 3111.21 with *I*^2^ = 99%), PCa patients recording an overweight condition, conversely, registered 0.98% of low-grade Gleason score rather than a high one [0.98 (0.77,1.25), *p* = 0.00] (Figure 4).

**Figure 5:**
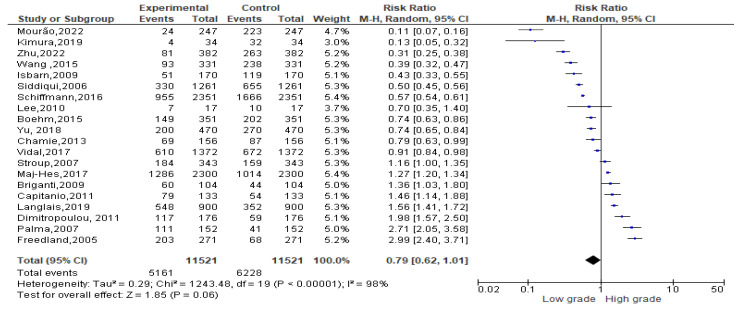
Gleason scores in obese PCa patients [34,37,38,39,40,41,47,50,52,53,54,55,56,58,59,60,61,62,63,64].

Among 11,389 obese events recorded, 5161 events belonged to a low-grade Gleason score and 6228 to a high-grade Gleason score. Since the Cochrane Q test showed high heterogeneity (*Tau*^2^ = 0.29 and *Chi*^2^ = 1243.48 with *I*^2^ = 98%), PCa patients recording an obesity condition registered 0.79% of low-grade Gleason score rather than a high one [0.79 (0.62, 1.01), *p* = 0.00] (Figure 5).

### 3.3. Meta-Analysis According to TNM Scores

**Figure 6:**
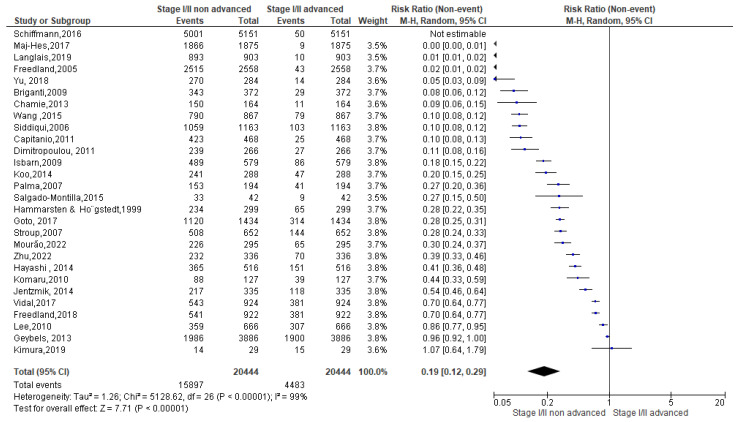
TNM scores in normal weight PCa patients [37,38,39,40,41,42,43,44,45,46,47,48,49,50,51,52,53,54,55,56,57,58,59,60,61,62,63,64].

A total of 61,202 events were registered. Of these, 20,444 reported a normal weight condition, 28,285 an overweight one and 12,473 an obese condition. Among normal weight events recorded (Figure 6), 15,897 events belonged to non-advanced TNM score and 4483 to advanced TNM score. Since the Cochrane Q test showed high heterogeneity (Tau^2^ = 1.26 and *Chi*^2^ = 5128.62 with *I*^2^ = 99%), PCa patients recording normal weight condition registered a significant incidence in non-advanced TNM score in their PCa conditions [0.19 (0.12, 0.29), *p* = 0.00] (Figure 6).

**Figure 7:**
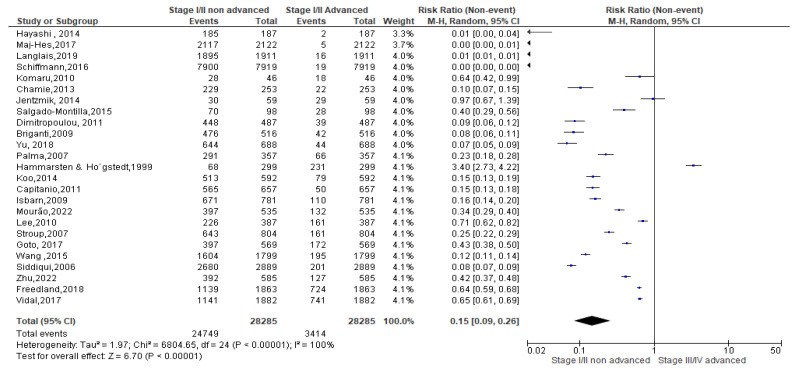
TNM scores in overweight PCa patients [37,38,39,40,42,44,45,46,47,48,50,51,52,53,54,55,56,57,58,59,60,61,62,63,64].

As regards the overweight condition, a total of 28,285 events were registered. Of these, 24,749 events belonged to non-advanced TNM score and 3414 to advanced TNM score. Since the Cochrane Q test showed high heterogeneity (Tau^2^ = 1.97 and *Chi*^2^ = 6804.65 with *I*^2^ = 100%), overweight PCa patients registered a significant incidence in non-advanced TNM score in their PCa conditions [0.15(0.09, 0.26), *p* = 0.00] (Figure 7).

**Figure 8:**
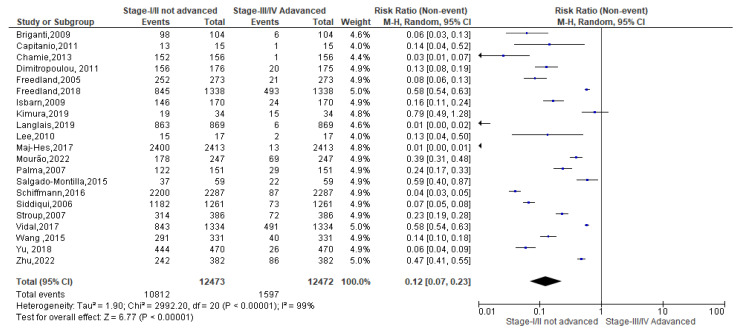
TNM scores in obese PCa patients [37,38,39,40,41,42,47,49,52,53,54,55,56,57,58,59,60,61,62,63,64].

Finally, as regards the obese condition, a total of 12,473 events were registered. Of these, 10,812 events belonged to non-advanced TNM score and 1597 to advanced TNM score. Since the Cochrane Q test showed high heterogeneity (Tau^2^ = 1.90 and *Chi*^2^ = 2992.20 with *I*^2^ = 99%), obese PCa patients registered a significant incidence in non-advanced TNM score in their PCa conditions [0.19 (0.07, 0.23), *p* = 0.00] (Figure 8).

## 4. Discussion

The present systematic review and meta-analyses aimed to highlight any existing trends between prostate neoplasm stages according to normal weight, overweight and obesity conditions. As regards the GS scoring system, 1.09% of high-grade GS was reported among PCa normal weights. Conversely, among PCa with overweight, it was 0.98% of low-grade GS was registered. The same trend was recorded among obese PCa patients, since 0.79% of low-grade GS was also registered. In this regard, evidence confirmed the same trend between BMI scores and GS ones, as a direct association was observed between BMI and the GS scores, specifically between higher BMI values and higher GS ones and also between normal BMI scores and low GS ones. These trends were comparable to data reported by Gioia et al. [65] among Caucasian men, and Liang et al. [5] in a cohort study assessing Selenium and Vitamin E for PCa prevention (SELECT), suggesting a relationship between higher BMI values and higher PCa grade risks, more specifically among men with a PCa family history. Obesity was also suggested as a likely risk factor for aggressive PCa and tumor recurrence [11] and race and high BMI were considered as independent of PCa’s high grade. These findings were also confirmed among Americans [11], wherein 72.4% of the patients with higher GS values were associated with higher BMI values, as also suggested by Zhou et al. [12] and by Kryvenko et al. [13], in which nearly 5118 PCa patients recorded higher GS values and also higher BMI ones. Additionally, Amling et al. [15] explained the impact of obesity on men undergoing radical prostatectomy, who reported the BMI variable as independent from GS scores and biochemical recurrence. Additional studies agreed to associate higher BMI values to worse prostate biopsy [1,11], while no associations were reported between obesity and PCa among European men [47,65].

In this regard, the PCa risk associated with higher BMI values might depend on different countries of origin and there was a lack of available evidence to consider the relationship between BMI and PCa growth and progression [16]. On the other hand, further evidence suggested that BMI was not associated with a raised risk of PCa at biopsy [66]. However, these results needed to be confirmed with additional studies among comparable populations also considering additional elements, such as the patient’s behaviors and serum hormone levels, especially in testosterone linked to obesity, which could favor the growth and progression of PCa aggressiveness [21]. As regards TNM scores, both normal weight, overweight and obese PCa patients registered a significant incidence in non-advanced TNM score, without any significant differences considering higher TNM assessments (Figure 6, Figure 7 and Figure 8). However, the literature showed discordant evidence in this aspect, as Harrison et al. [22] observed no associations between BMI and PCa risk, which seemed to be in contrast to our present findings. However, Cao and Ma [24] underlined high heterogeneity among the reviewed studies and additional stratification of their data suggested a positive association between BMI and PCa mortality rates. These findings were consistent with those reported in another meta-analysis [19], which highlighted an inverse association between higher BMI scores and localized PCa and a positive association between higher BMI scores and advanced PCa. Thus, obese PCa men seemed to be more likely to develop an aggressive PCa with also a high tumor volume. Additionally, obese PCa men reported a significantly higher risk of disease recurrence, as well as an increase in mortality rate compared to normal weight PCa men [24]. On the other hand, previous analyses have suggested that obesity may not significantly influence the risk or aggressiveness of PCa, or that the association could depend on other factors, like hormonal status, race and timing of diagnosis [67]. Obese patients, although not presenting with more extensive disease initially, could have more aggressive histologic tumors. Thus, obesity could be considered a risk factor for high-grade tumors, even if not for greater local extension [68]. In this regard, the low androgen microenvironment in obese men could play an important role, but further studies will be necessary in this direction [28,29,30].

Eating lifestyles played a pivotal role in PCa biology and its related etiopathogenesis, on both the growth and progression of PCa. On the other hand, numerous nutrients and herbs played a promising role in decelerating PCa progression and decreasing the risk of morbidity and mortality, also adding benefits for the cardiovascular system, bone health and for the prevention of other cancers [69].

However, no specific mechanisms were highlighted between obesity and PCa progression [48], despite hyperinsulinemia, higher levels of growth factors, inflammation and its related cytokines and chemokines, alterations in steroid hormones and adiponectin levels and other factors (dysfunctions in microbiome, angiogenesis) have been mentioned [70,71]. In the obesity condition, especially in abdominal obesity, an increase in white adipose tissue (WAT) has been highlighted and several studies supported the linkage between PCa progression in the excessive presence of WAT [72,73]. Additionally, increased WAT concentrations could boost a latent chronic inflammation condition, known as an inducing role in the obesity–cancer relationship [74], also due to the interaction with cancer cells inducing cancer associated with adipocytes and contributing to the development of aggressive PCa [75]. Hence, the present systematic review and meta-analysis highlighted important implications in BMI in PCa staging, while at the same time, had some limitations. First of all, data included several study designs and it could be helpful to have a major number of cases. However, this methodological choice could negatively impact the quality of evidence or the comparability of data, due to the very high heterogeneity among the included data and the lack of adjustment for potential confounding factors.

A limitation of this meta-analysis is the use of BMI alone as a proxy for overweight and obesity. Although BMI is a valuable tool for population surveys and primary care screening, it has limitations when used as the sole tool for predicting chronic disease risk and assessing excess fat. BMI cut-off values should be reconsidered in populations of varying body build, age and/or ethnicity. Given that overweight individuals diagnosed by BMI are sometimes physically and physiologically fit by other measures, individuals overweight by, e.g., BMI should be more comprehensively assessed, diagnosed and monitored with anthropometric parameters relating to the amount of lean mass and fat mass adjusted for age, height and ethnicity. We have not evaluated the effect of being “underweight” on PCa prevalence; however, a meta-analysis including fewer studies (13 studies) reported that underweight PCa patients exhibited a tendency to present a decreased risk of PCa compared to those with normal weight; however, the effect was not statistically significant [76].

## 5. Conclusions

Obesity has been associated with a 20–30% increased risk of PCa death. The present systematic review and meta-analyses aimed to highlight any existing trends between prostate neoplasm stages (Gleason score, GS) according to normal weight, overweight and obesity conditions. As regards the GS scoring system, 1.09% of high-grade GS was reported among PCa normal weights. Among overweight PCa, a 0.98% of low-grade GS was registered. The same trend was recorded among obese PCa patients, since 0.79% of low-grade GS was also registered. Future studies, preferably prospective observational studies or patient-level pooled analyses, will investigate in greater depth the relationship between obesity and prostate cancer aggressiveness, ideally incorporating biological variables such as hormonal markers and inflammation.

## Figures and Tables

**Table 1:** Search strings carried out to perform this systematic and meta-analysis study.

Database	Results
**Embase** (‘association’/exp OR ‘association’) AND (‘body mass index’/exp OR ‘body mass index’) AND (‘neoplasm staging’/exp OR ‘neoplasm staging’) AND (‘prostatic neoplasms’/exp OR ‘prostatic neoplasms’)	457
**PubMed** (((Association) AND (Body Mass Index)) AND (Neoplasm Staging)) AND (Prostatic Neoplasms) (“associate”[All Fields] OR “associated”[All Fields] OR “associates”[All Fields] OR “associating”[All Fields] OR “association”[MeSH Terms] OR “association”[All Fields] OR “associations”[All Fields]) AND (“body mass index”[MeSH Terms] OR (“body”[All Fields] AND “mass”[All Fields] AND “index”[All Fields]) OR “body mass index”[All Fields]) AND (“neoplasm staging”[MeSH Terms] OR (“neoplasm”[All Fields] AND “staging”[All Fields]) OR “neoplasm staging”[All Fields]) AND (“prostatic neoplasms”[MeSH Terms] OR (“prostatic”[All Fields] AND “neoplasms”[All Fields]) OR “prostatic neoplasms”[All Fields])Translations Association: “associate”[All Fields] OR “associated”[All Fields] OR “associates”[All Fields] OR “associating”[All Fields] OR “association”[MeSH Terms] OR “association”[All Fields] OR “associations”[All Fields]Body Mass Index: “body mass index”[MeSH Terms] OR (“body”[All Fields] AND “mass”[All Fields] AND “index”[All Fields]) OR “body mass index”[All Fields]Neoplasm Staging: “neoplasm staging”[MeSH Terms] OR (“neoplasm”[All Fields] AND “staging”[All Fields]) OR “neoplasm staging”[All Fields]Prostatic Neoplasms: “prostatic neoplasms”[MeSH Terms] OR (“prostatic”[All Fields] AND “neoplasms”[All Fields]) OR “prostatic neoplasms”[All Fields]	112
**Scopus** (TITLE-ABS-KEY (association) AND TITLE-ABS-KEY (body AND mass AND index) AND TITLE-ABS-KEY (neoplasm AND staging) AND TITLE-ABS-KEY (prostatic AND neoplasms))	211
**WoS** Association (All Fields) and Body Mass Index (All Fields) and Neoplasm Staging (All Fields) and Prostatic Neoplasms (All Fields)	32

**Table 2:** The PIO tool for the present systematic review and meta-analysis.

**Population**	Prostate Cancer Patients
**Intervention**	Prostate cancer staged according to Gleason or TNM scores
**Outcome**	Incidence of normal weight, overweight and obese prostate cancer patients

**Table 3:** The main characteristics of the studies included in the present systematic review and meta-analysis (n = 29).

Author(s) Publication Year	Study Design GS or/and TNM Score	Aim	Sample Size	Findings
Boehm et al., 2015 [34]	Case-control study GS	To assess any associations between WC, waist–hip ratio, BMI and PCa risk.	1933 PCa men diagnosed between 2005 and 2009	Higher BMI scores were associated with lower PCs risk. Abdominal obesity had an inverse trend.
Briganti et al., 2009 [37]	Observational and retrospective study GS and TNM	To assess any associations between obesity and PCa aggressiveness.	994 PCa patients	Greater BMI levels were not associated with increased risk of PCa aggressiveness.
Capitanio et al., 2011 [38]	Prognosis study GS and TNM	To assess any associations between BMI and tumor volume.	1275 PCa patients undergo RP	BMI seemed to be independent of prostate cancer volume at RP. BMI might play a key role in PCa pathophysiology.
Charmie et al., 2013 [39]	Retrospective study GS and TNM	To examine associations between obesity and PCa clinical stage.	573 PCa patients	BMI did not impact on the interpretation of pre-biopsy PSA levels in those with PCa cancer.
Dimitropoulou et al., 2011 [40]		To examine the association between obesity and the increased prostate cancer risk.	11368 PSA cases between 2001 and 2008	BMI was associated with a decreased risk of low-grade PSA-detected prostate cancer.
Freedland et al., 2005 [41]	Prospective cohort study GS and TNM	To investigate any associations between obesity and PCa staging.	2832 PCa men were recruited between 1985 and 2004	The positive association between obesity and high-grade disease, and BCR after radical RP was strongest among treated men.
Freedland et al., 2019 [42]	Prospective cohort study GS and TNM	To investigate any associations between BMI and BCR.	4132 PCa patients between 1985 and 2015	Greater BMI was associated with BCR.
Geybels et al., 2013 [43]	Observational and retrospective study TNM	To analyze any associations between flavonoid and black tea assumptions, and PCa risk.	3362 PCa men diagnosed from 1986 to 2003	Flavonoid and black tea assumptions consumption were associated with a decreased risk of greater PCa stage.
Goto et al., 2017 [44]	Observational and retrospective study GS and TNM	To assess any associations between BMI and the clinic-pathological features in PCa patients.	2003 Japanese patients eligible for radical prostatectomy	A significant association between higher BMI and higher Gleason score were recorded. BMI values may be considered as a potential classifier for predicting adverse pathological occurrences.
Hammarsten and Högstedt 1999 [45]	Observational and retrospective study TNM	To explore the existing linkage between PCa and MetS.	299 PCa patients	PCa was recognized as a component of the MetS with abnormalities both in insulin-mediated glucose uptake and hyperinsulinaemia.
Hayashi et al., 2014 [46]	Observational and retrospective study TNM	To assess the impact of BMI on BCR after RP for PCa in Japanese men.	3362 PCa men diagnosed from 2002 to 2009	Higher BMI scores were associated with higher BCR rates. BMI values were associated with high ITV grade. Greater BMI may contribute to increasing tumor staging.
Isbarn et al., 2009 [47]	Observational and retrospective study GS and TNM	To assess BMI values as predictors of PCa advanced stages in RP patients.	1538 PCa patients recruited from 2005 to 2008	Obese patients were not associated with PCa stages.
Jentzmik et al., 2014 [48]	Observational and retrospective study GS and TNM	To assess any associations between testosterone levels, obesity and tumor stage/grade.	510 European Caucasian men	Low serum testosterone concentrations were significantly associated with high-grade and metastatic PCa.
Kimura et al., 2019 [49]	Cross-sectional observational study GS and TNM	To investigate the presence of sarcopenia and sarcopenic obesity in PCa older men treated with ADT.	89 PCa patients treated with ADT	Sarcopenic obesity.
Komaru et al., 2010 [50]	Observational and retrospective study GS and TNM	To assess any associations between obesity and PCa.	173 PCa men treated with RP from 1997 to 2007	Obesity appeared to be an independent predictor of disease recurrence.
Koo et al., 2014 [51]	Observational and retrospective study GS and TNM	To investigate the impact of obesity on BCR in Korean PCa patients treated with RP.	880 PCa patients between 2005 and 2011.	Obese and overweight Korean PCa patients reported lower GS values and a reduced risk of BCR than normal weight counterparts.
Langlais et al., 2020 [52]	Observational study GS and TNM	To assess any associations between BMI and prognostic risk in PCa.	5200 PCa patients recruited from 1995 to 2017	Normal weight might improve OS.
Lee et al., 2010 [53]	Case series GS and TNM	To assess the impact of BMI on pathological features after RP in Korean patients.	1000 Korean patients	Greater BMI scores were significantly associated with extracapsular extension of PCa. However, BMI did not preoperatively predict tumor extension.
Maj-Hes et al., 2017 [54]	Validation study GS and TNM	To assess any associations between BMI and outcomes after RP.	6519 RP PCa patients	Overweight and obese conditions were associated with BCR after RP.
Mourao et al., 2022 [55]	Observational and retrospective study GS and TNM	To assess any associations between obesity and urinary incontinence.	1077 men eligible for RARP	Obese safely underwent RARP with comparable continence goals than to non-obese men.
Palma et al., 2007 [56]	Observational and retrospective study GS and TNM	To assess whether obesity was associated with adverse disease features, pre-treatment serum testosterone, bDFS, DSS or OS in PCa patients.	909 PCa men were enrolled between 1994 and 2001	Obesity was associated with lower serum testosterone concentrations and a higher risk of recurrence and prostate cancer-specific death after RT.
Salgado-Montilla et al., 2015 [57]	Retrospective medical record review study TNM	To investigate any associations between lipid concentrations and PCa phenotype.	199 PCa patients undergo RP	Higher levels in triglycerides seemed to be associated with PCa phenotype and growth.
Schiffmann et al., 2017 [58]	Retrospective medical record review study GS and TNM	To investigate the impact of obesity on BCR in PCa patients.	16,014 PCa men were enrolled between 2004 and 2015	Obesity could induce a higher response to non-organ-confined PCa.
Siddiqui et al., 2006 [59]	Prospective cohort study GS and TNM	To assess the impact of obesity on PCa long-term outcomes.	5313 men undergo RP from 1990 to 1999	BMI impacted PCa outcomes at RP, but after RP, BMI did not appear to be an independent predictor of recurrence or survival.
Stroup et al., 2007 [60]	Retrospective cohort study GS and TNM	To assess the effect of obesity on BCR after EBRT.	1868 PCa patients recruited from 1989 to 2003	Higher BMI scores were associated with higher odds of BCR.
Vidal et al., 2017 [61]	Retrospective cohort study GS and TNM	To investigate any associations between obesity and long-term PCSM.	4268 PCa patients undergo RP	Overweight and obesity were associated with increase in PCSM after RP.
Wang et al., 2015 [62]	Retrospective cohort study GS and TNM	To examine any associations between BMI and RT.	1442 PCa patients recruited from 2001 to 2010	Higher BMI scores seemed to be associated with increased levels of BF, DM and CSM.
Yu et al., 2018 [63]	Retrospective cohort study GS and TNM	To assess any associations between BMI and BC in RP patients.	2997 PCa patients after RP	Obese patients were more predisposed to have lower BCR-free-survival.
Zhu et al., 2022 [64]	Observational and retrospective study GS and TNM	To investigate any effects of BMI and DM in prostate cancer (PCa) risk groups.	1303 PCa men	A supplemental effect was recorded in obesity and DM in PCa risk.

Abbreviations: ADT: androgen deprivation therapy; BCR: biochemical recurrence; BF: biochemical failure; bDFS: biochemical disease-free survival; BMI: body mass index; CSM: cause-specific mortality; DM: diabetes mellitus; DM: distant metastases; DSS: disease-specific survival; EBRT: external beam radiation therapy; GS: Gleason score; ITV: index tumor volume; MetS: metabolic syndrome; OS: overall survival; PCa: prostate cancer; PCSM: prostate cancer-specific mortality; RARP: robot-assisted radical prostatectomy; RP: radical prostatectomy; RT: radiation therapy; TNM: TNM classification of malignant tumors; WC: waist circumference.

## Data Availability

Data are available from first co-authors upon reasonable request.
